# Technology Acceptance and Usability of a Therapy System with a Humanoid Robot Serving as Therapeutic Assistant for Post-Stroke Arm and Neurovisual Rehabilitation—An Evaluation Based on Stroke Survivors’ Experience

**DOI:** 10.3390/biomimetics10050289

**Published:** 2025-05-04

**Authors:** Thomas Platz, Alexandru-Nicolae Umlauft, Ann Louise Pedersen, Peter Forbrig

**Affiliations:** 1 Neurorehabilitation Research Group, University Medical Centre, 17475 Greifswald, Germany; 2 BDH-Klinik Greifswald, Institute for Neurorehabilitation and Evidence-Based Practice (“An-Institut”), University of Greifswald, 17491 Greifswald, Germany; 3 Department of Computer Science, University of Rostock, 18059 Rostock, Germany

**Keywords:** robot training, arm neglect, stroke acceptance, usability technology, social artificial intelligence

## Abstract

**Background:** This study performed an evaluation of technology acceptance of the therapeutic system E-BRAiN (Evidence-Based Robot Assistance in Neurorehabilitation) by stroke survivors receiving therapy with the system. **Methods:** The evaluation was based on a 49-item questionnaire addressing technology acceptance (I) with its constituents, i.e., perceived usefulness, perceived ease of use, perceived adaptability, perceived enjoyment, attitude, trust, anxiety, social influence, perceived sociability, and social presence (41 items), and (II) more general items exploring user experience in terms of both technology acceptance (3 items) and usability (5 open-question items). **Results:** Eleven consecutive sub-acute stroke survivors who had received either arm rehabilitation sessions (n = 5) or neglect therapy (n = 6) led by a humanoid robot participated. The multidimensional “strength of acceptance” summary statistic (Part I) indicates a high degree of technology acceptance (mean, 4.0; 95% CI, 3.7 to 4.3), as does the “general acceptance” summary statistic (mean, 4.1; 95% CI, 3.3 to 4.9) (art II) (scores ranging from 1, lowest degree of acceptance, to 5, highest degree of acceptance, with a score of 3 as neutral experience anchor). Positive ratings were also documented for all assessed constituents (Part I), as well as the perception that it makes sense to use the robot technology for stroke therapy and as a supplement for users’ own therapy (Part II). **Conclusions:** A high degree of technology acceptance and its constituents, i.e., perceived functionality and social behaviour of the humanoid robot and own emotions while using the system, could be corroborated among stroke survivors who used the therapeutic system E-BRAiN.

## 1. Introduction

Neurological diseases are the leading causes of disability relevant to everyday life worldwide [1]. Although acute medicine is becoming ever more efficient and successful thanks to scientific progress, demographic changes with an ageing population, as well as unfavourable lifestyle factors and behaviour, are making neurological diseases and the resulting everyday disabilities increasingly common in our societies. Among neurological diseases, stroke is the most common cause of disability.

Neurorehabilitation helps reduce stroke-related disabilities, enabling more individuals to regain independence and care for themselves [2,3]. This success is due to the brain’s ability to reorganize and recover functionally [4]. Recovery can occur spontaneously but is enhanced by targeted, intensive training, known as “neural repair therapy” [5]. Research shows that specific repetitive training protocols for targeted impairments are more effective than conventional therapy, even with the same amount of time [6]. Despite being recommended by international guidelines [7], such therapy is often underused due to a shortage of skilled therapists, particularly in low- and middle-income countries [8]. Thus, there is a growing need for more intensive and specific neurorehabilitation services that is difficult to meet due to capacity limitations of the qualified human workforce.

Technology that focuses on a single aspect, like digital health apps with training schedules, do not provide a social context and interactions that are frequently necessary to support training behaviour. Accordingly, apps may not fully meet the needs of individuals with neuro-disabilities requiring restorative training. Therapy systems making use of socially interactive robots offer a more comprehensive solution, addressing the interpersonal side of therapy. While still an emerging technology, first research shows that stroke survivors can benefit from rehabilitation with humanoid robots that enhance therapies beyond traditional computer interfaces [9,10].

The E-BRAiN system (Evidence-Based Robot-Assistance in Neurorehabilitation (https://www.ebrain-science.de/en/home/; accessed on 7 March 2025)) used in this research employs a humanoid robot to guide stroke survivors through therapy sessions, offering instructions, feedback, and motivation [11,12]. It is conceptualised to cover the therapeutic interactions needed comprehensively. Before autonomous robot-led therapy sessions are commenced, they are individualised. Shared therapeutic decisions are taken between physician, therapist in charge, and the stroke survivor. A human therapist then personalises the prescribed training before training with the humanoid robots commences. The system then autonomously leads daily sessions based on evidence-based therapies, such as arm rehabilitation and neglect therapy, and adapts its activities based on individual data like clinical assessments, goals, individualised training specifications, and therapeutic progress made. It also generates real-time text responses tailored to the user’s needs [13]. Overall, the consecutive robot-led therapeutic sessions are conceptualised to be run without direct involvement of a qualified human therapist.

To reach this goal of innovation, i.e., the autonomous therapy administration by a system making use of a humanoid robot, the E-BRAiN system combines therapeutic knowledge, individualized training, feedback, and social interaction, making it a comprehensive tool for stroke rehabilitation. Therapeutic knowledge is embedded in algorithms that contain the tasks for each type of training implemented (for details, see Section 2.7, “Description of the Therapies Implemented”), their specific sequence and related audiovisual presentations, e.g., photos and videos with a model performing the training tasks or presentation of training task stimuli on a large computer screen (for neurovisual therapy). From these libraries, the appropriate selection and parametrisation for individual patients is supported by a database that hosts individualised clinical information for its use by the system (e.g., clinical assessment, therapeutic goal, and training data). During training sessions, the therapeutic dialogue merges standardised therapy information and individualised content, providing both more general information regarding the training and its relevance for an individual’s goal, specific audio-visually supported instructions for each training task, and individualised feedback (e.g., as intermittent knowledge of results with both a graphical display and graded natural speech comments) in accordance with the individual’s parametrisations made. Such therapeutic dialogue is based on a conceptualisation that includes information provision, feedback, and bonding and has been designed to mimic human therapists that administer the same type of therapy to stroke survivors [11]. Indeed, it has been documented that the system reliably provides therapeutic interaction that closely resembles human interaction and varies in accordance with the affordances of different types of therapy and a course of consecutive training sessions [12]. Thereby, the E-BRAiN system reaches a high level of comprehensiveness for autonomous therapy administration across different types of stroke rehabilitation therapy.

While it is good scientific practice to evaluate new therapeutic methods with adequate clinical trials that assess both clinical benefits; patient-reported outcome; and any potential harm, e.g., by randomised clinical trials, another important aspect of evaluation for emerging technologies is to assess how acceptable a technology is for users and how they perceive the usability of a technical system.

Testing subjective user impressions of aspects like ease to use or adequacy of the support received by technology are crucial for the development of user-friendly digital health applications to promote user acceptance, efficient patient management, and, in consequence, the intended improved health outcomes [14].

User-friendliness is part of an acceptance system and the resulting intention to use a system. For social robot systems, system acceptance can be divided into two aspects, namely acceptance of the robot in terms of usefulness and ease of use (functional acceptance), and acceptance of the robot as a conversational partner with which a human-like relationship is possible (social acceptance) [15].

According to theoretical models like the Unified Theory of Acceptance and Use of Technology (UTAUT) [16], acceptance and use of technology are determined by a variety of factors. More recently, a refined model (called the Almere model) has been proposed that integrates several partially interrelated factors that together influence technology acceptance [15]. These factors are perceived usefulness, perceived ease of use, perceived adaptability, perceived enjoyment, attitude, trust, anxiety, social influence, perceived sociability, and social presence (for an explanation of these factors, see Table 1 below). Technology acceptance, again, is important, as it determines the “intention to use”, i.e., the resultant subjective construct reflecting the likelihood of the future use of a technology. For rehabilitation, where intensive training schedules need to be followed for prolonged periods, technology acceptance is key for the provision of and adherence to technology-supported healthcare.

Models like the Almere model point to a complex information structure to be assessed to arrive at measures that represent both functional and social acceptance.

At the same time, less complex methods such as expert reviews or rapid iterative testing and evaluation methods are mostly recommended for the evaluation of usability of eHealth applications to promote efficiency for product development improvement cycles [17].

To maintain a balance, this study aimed for both a theory-based and easy-to-use technology acceptance evaluation for the digitally implemented therapeutic system E-BRAiN.

For this purpose, this study used the theory-based questionnaire developed by Heerink et al. [15], addressing acceptance and “intention to use”, and we added a few questions addressing the system usability.

## 2. Methods

### 2.1. Study Type

Observational study planned as a first assessment of acceptance and usability of the therapeutic system E-BRAiN (Evidence-Based Robot Assistance in Neurorehabilitation) as experienced by stroke survivors receiving therapy with the system.

The observational study is an associated project with the clinical trial Evidence-based Robot Assistant in Neurorehabilitation (https://clinicaltrials.gov/ct2/show/NCT05152433; accessed on 7 March 2025) [18].

### 2.2. Recruitment of Participants

Participants were stroke survivors participating in the clinical trial Evidence-Based Robot Assistant in Neurorehabilitation [18] who were willing to participate in the additional assessment of acceptance and usability of the therapeutic system E-BRAiN during their phase of robot training while participating in the clinical trial. Eligible were stroke survivors with either arm paresis or visual neglect who agreed to receive robot therapy with the E-BRAiN system.

A total of 11 consecutive patients were recruited for the assessment of acceptance and usability, receiving diverse forms of training-based therapy (across subjects), as offered by the digital therapy system (i.e., arm basis training, mirror therapy, and neglect therapy).

### 2.3. Participant Characteristics

At the time of study enrolment, the following characteristics were recorded for all participants: age, gender, type of stroke aetiology (ischemic stroke, non-traumatic intracerebral haemorrhage, or subarachnoid haemorrhage), time since the stroke (in weeks), degree of neuro-impairment (measured using the National Institute of Health Stroke Scale, NIHSS) [19], degree of neuro-disability (assessed with the Barthel Index) [20], arm motor function (Fugl–Meyer Arm Motor score; FM Arm for participants with moderate-to-severe arm paresis) [21], or visual neglect (neglect test, NET, for participants with visual neglect) [22].

### 2.4. Questionnaire

All participants received a questionnaire (“Fragebogen zur Einschätzung des Therapiesystems durch Nutzer” (translation: Questionnaire for the assessment of the therapy system by users)) that had been purpose built.

The 5-page questionnaire has two parts.

Part I includes 41 items (as published by Heerink et al. [15]) addressing aspects of technology acceptance, each coded as a 5-point Likert scale ranging from “do not agree at all” (German: “stimme überhaupt nicht zu”) to “completely agree” (German: “stimme komplett zu”).

Part II constituted (a) 3 items addressing more general aspects of technology acceptance for the E-BRAiN system, again each coded with the same 5-point Likert scale; and (b) 5 open questions addressing the system’s usability (subjective user experience).

For items in Part I, Table 1 presents the constructs assessed and their definition, and Table 2 presents the statements (items) used to assess these constructs, as well as each individual item’s mapping to one of the twelve constructs assessed [15]. The statements were translated to German (by TP and ANU) for this study. The German questionnaire is provided in the Supplementary Materials.

In Part II, section a contains the following three more generally phrased Likert-scale items to assess technology acceptance for the E-BRAiN system: (1) “I think it’s a good idea to use a robot in stroke therapy” (“robot for stroke”). (2) “I think it makes sense to use this therapy with the robot as a supplement to my therapy” (“robot therapy for me”). (3) “A therapy session with the robot is fun” (“fun”). These questions were used to provide more “general” acceptance information compared to the 41 items of Part I of the questionnaire, while at the same time being specifically phrased for the E-BRAiN system. The intention was to generate acceptance data that are less diversified and less dependent on the validity of a complex model like the Almere model. Thereby, the results can be used to validate the more complex approach to acceptability profiling of Part I (“face validity”).

In section b of Part II, the following open questions addressing usability aspects were posed to participants: (1) What did you like best about this system (and your therapy)? (2) What did you like least about this system (and your therapy)? (3) If you could change or add one thing about this system, what would it be? (4) Now, please think about your overall experience with the E-BRAiN system: How did you find therapy with the robot? What do you think about the robot as a therapist? (5) Is there anything else you would like to tell us about the system?

### 2.5. Outcome Measures

Outcome measures of interest were as follows:

- Construct-related statistics, i.e., the average score of items mapping to one of the twelve constructs assessed (secondary variables), were used to generate a technology acceptance profile (resembling estimates for the twelve constructs assessed as based on the 41 items of Part I of the questionnaire);
- A grand average across all 41 items was used as an overall measure of technology acceptance, i.e., a summary multidimensional “strength of acceptance” statistic;
- Item statistics for the three more general technology acceptance items for the E-BRAiN system and their grand average statistic (based on an average of the three items of Part II, section a of the questionnaire) (Part II, section a);
- Qualitative summary of information documented as responses to the 5 open questions (Part II, section b): strengths of the systems as experienced by stroke survivors as users for their rehabilitation therapy, its weaknesses, areas where modifications were suggested by users, and overall users’ subjective experience.

### 2.6. Study Procedures

Participants of the clinical trial E-BRAiN received their therapy as inpatients (sub-acute rehabilitation) at the BDH-Klinik Greifswald, a university-affiliated neurorehabilitation centre, in regular rehabilitation therapy rooms. They were informed about the acceptance and usability evaluation and invited to participate, and they became participants of the technology acceptance and usability evaluation after granting written informed consent.

For these participants, a single session was scheduled for the administration of the questionnaire right after a therapy session with the robot system. The questionnaire administration was scheduled no earlier than after at least 3 training sessions with the E-BRAiN system to ensure user sufficient experience, and no later than 2 days before the final 9th session with the E-BRAiN session to guarantee further experience with the robot to come and, hence, a valid background to assess the intention to use the system again.

The questionnaire administration lasted about 1 to 1.5 h, was planned to fit within the participant’s daily schedule, and was conducted by ANU. To reduce barriers that were related to stroke-related impairments (including cognition), participants could receive help in case any question arose about the meaning of questionnaire items or how to provide questionnaire responses.

### 2.7. Description of the Therapies Implemented

The *arm basis training*, *ABT,* has been developed for stroke survivors with moderate-to-severe arm paresis and focuses on joint-movement training to promote the recovery of the ability to control movements in arm, hand, and finger joints selectively [6]. As stroke survivors with more severe paresis face difficulties moving their arm, hand, and fingers, the training movements are assisted by a therapist as needed. In ABT sessions with the humanoid robot, it is the robot that leads therapy sessions, providing interaction, while a helper (no qualified therapist required) may provide additional physical support during ABT when needed.

*Mirror therapy* for arm paresis post-stroke involves using a mirror to create the illusion that the affected arm is moving normally. The patient places the unaffected arm in front of a mirror while the affected arm is hidden, i.e., lying behind the mirror. As the unaffected arm moves, the reflection in the mirror makes it appear as though the affected arm is moving as well and does so normally. This visual feedback can promote motor recovery [23]. The E-BRAiN system applies mirror therapy with items of trained movements resembling movement tasks taken from the ABT (but performed with the non-affected arm and hand).

*Neurovisual therapy*, *NVT,* for visual neglect post-stroke focuses on improving attention and visual exploration in patients with hemineglect, a condition where one side of space is ignored. The NVT implemented in E-BRAiN includes optokinetic stimulation, where moving visual patterns toward the neglected hemispace helps activate the brain’s networks for spatial attention; saccadic eye movement training, which encourages the patient to shift their gaze toward a single stimulus, including into neglected areas; and visual exploration training, which encourages scanning and exploring complex visual pattern presented across the visual field, including neglected parts [24]. These techniques work together to enhance spatial attention and support recovery by retraining the brain to properly attend to and shift attention to either side of the visual field within the context of specific tasks.

### 2.8. Description of the Digital Therapy System E-BRAiN

**Figure 1:**
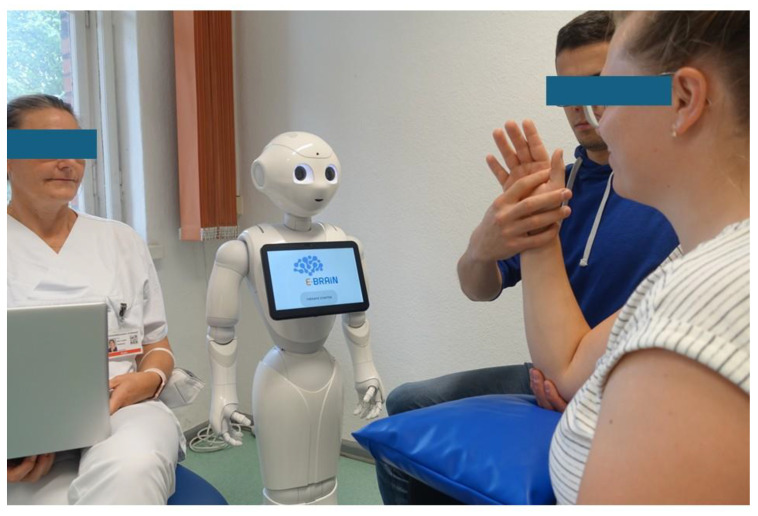
The figure depicts a therapeutic scenario for the digital therapeutic system E-BRAiN, which uses a humanoid robot to facilitate therapeutic interactions during arm rehabilitation or neurovisual therapy sessions for stroke survivors. Here, the scenario for the arm basis training (ABT) for moderate-to-severe arm paresis is shown. The robot delivers all therapeutic interactions (such as providing information, feedback, and engagement) while guiding the patient through a series of standardised, yet individually prescribed training tasks. An additional helper—someone not qualified as a therapist—joins as a second active human participant. This person, also guided by the humanoid robot, offers physical assistance as needed for the conduct of training tasks that patients with a severely paretic arm cannot perform by themselves. For study purposes, a supervising staff member is present in the background (not as prominent as depicted in the photo for the photographer’s convenience), monitoring the process and ready to intervene if the autonomous system encounters an error or if the patient requires assistance that the system cannot provide.

The robot system, shown in Figure 1, consists of interconnected, devices including a humanoid robot (Pepper; https://www.unitedrobotics.group/products-services/hardware/; accessed on 7 March 2025), tablet, and touch monitor, all controlled via a central computer running CentOS Linux 7 (Core) (Linux operating system; https://www.linux.org/; accessed on 7 March 2025). The system uses MQTT v3.1.1 (https://mqtt.org/; accessed on 7 March 2025) messaging for device communication and is managed by therapists through an interface.

The digital therapy system E-BRAiN makes use of humanoid robot technology to serve as a social agent. Its role is to provide all therapeutic interaction necessary for (comprehensive) therapy administration. It does, however, not provide physical assistance. This makes it distinct from other currently available robotic rehabilitation technology that gives mechanical support without providing social interaction [25].

During therapeutic sessions, the robot was in charge to provide all therapeutic interactions by verbal dialogue, including explanations of the therapy and its mechanisms of action, instructions, feedback as knowledge of performance or result, and addressing personal needs. For these purposes, the system displayed images, videos, and verbal information both spoken aloud by the robot and shown on a screen (e.g., for instructions); provided diagrams (e.g., for feedback on results); and asked for patient input (e.g., “do you need a break?” or “ready to continue?”). A touch monitor, with its 27-inch screen, was used for the neurovisual therapy (not shown in Figure 1).

Overall, the system supports intensive stroke rehabilitation with personalised information, instructions, feedback, and motivation.

In case physical help is needed for the conduct of training tasks (as is the case for the ABT), a helper is integrated into the training situation; such a “helper” is not a qualified therapist (e.g., could be a relative), so the helper, together with the stroke survivor, receives instructions from the system.

Technically, a “finite-state machine” design allows for precise control over therapy progression, with flexible pauses and transitions between states [26]. For that purpose, the robot operates within predefined therapy “states”, each representing a segment of the therapy program. Starting at the “start” state, the robot proceeds to the next state after a set time or upon patient confirmation, continuing until the final “saying goodbye” state is reached. Each therapy state is associated with media content and robot actions to be executed when that state is active. When the script moves to a new state, a message is sent to all connected devices (robot, tablet, or monitor). These devices interpret the message and execute commands, like displaying videos or providing speech feedback. This design allows flexible control of the robot, enabling it to pause at any therapy state and resume or switch to other states as needed, ensuring the patient follows the predefined content in the correct order.

While the digital therapy system E-BRAiN uses algorithms that are individually adapted both prior to its use for individuals (i.e., by individualised adaptations of standardised training schemas by the supervising staff) and based on data collected during therapy sessions, all decisions taken by the system are predefined by algorithms and do not rest on machine learning.

Although the system is conceptualised and administers therapeutic sessions autonomously, a staff member supervised the sessions during our research in case the need for interaction arose. A system programmer from the technical team (ANU) had remote access to monitor the system for errors and provide any fixes if needed.

### 2.9. Statistical Analyses

*Participants*

The group of participants was described by sociodemographic and stroke-related characteristics, and the therapy received (mean/SD or count/relative frequency as appropriate).

*Questionnaire*

All Likert-scale variables were coded numerically, with “1” indicating strong disagreement (“do not agree at all” (“stimme überhaupt nicht zu”)), and “5” indicating strong agreement (“completely agree” (“stimme komplett zu”)). For most items, higher scores indicated a subjective experience increasing the degree of acceptance. As some items had a revers association to acceptance (i.e., higher scores indicating a subjective experience decreasing the degree of acceptance), such items had been recoded so that all item scores indicated a subjective experience increasing the likelihood of acceptance with higher scores.

For Part I of the questionnaire, the mean and 95% confidence intervals for the construct-related statistics (secondary variables) and the summary multidimensional “strength of acceptance” were used to generate a technology acceptance profile (resembling estimates for the constructs assessed).

Similarly, for Part II of the questionnaire, both the variables from the 3 items using a Likert scale and their grand average were analysed using descriptive statistics, i.e., mean and 95% confidence intervals.

*Multivariate Analysis of Modifiers of Acceptance*

With the restrictions given by the small study sample, the data were explored (hypothesis generating) as to which clinical variables, i.e., age, sex, time post stroke, syndrome treated (arm paresis or visual neglect), general post-stroke neurological impairment (NIHSS scores), severity of impairment of the syndrome treated (FM Arm or NET scores), and disability (BI scores), modified acceptance. For this purpose, analyses of variance (ANOVAs) were performed using general linear models (GLMs) with either of the two summary acceptance statistics, i.e., the “strength of acceptance” or the “general acceptance” statistic as dependent variable, and age, sex, time post-stroke, severity of impairment, and disability served as independent variables. In case the model indicated statistically significant effects (*p* < 0.05), type III sums of squares were used to assess the relevance of individual independent factors as modifiers statistically.

## 3. Results

### 3.1. Participants

Data of the eleven stroke survivors receiving either arm rehabilitation sessions (i.e., ABT [n = 4] or MT [n = 1]) or neglect therapy (NVT [n = 6]) using the humanoid robot as therapeutic agent over a course of nine sessions were used for the purpose of this study.

The group of participants (compare Table 3) shows a considerable age distribution; (by chance) had mainly male participants; had suffered from an ischemic stroke in the sub-acute phase; had moderate neuro-impairment (NIHSS) [19] and neuro-disability (Barthel Index) [20]; and, within the subgroups, had either severe arm paresis (compare FM Arm scores, respectively) [21] or mild-to-severe visual neglect (compare NET scores) [22].

### 3.2. Acceptance Profile

Table 4 provides the results for the acceptance profile.

In Part I, the average statistics across items that individually range from 1 to 5 (low to high acceptability) and belong to a certain construct are presented together with a grand average score across all other construct statistics of Part I, i.e., a multidimensional “strength of acceptance” summary statistic.

As a score of 3 (“I don’t know” (“ich weiß nicht”)) is neutral, any statistics for which both the mean and 95% confidence interval are above 3 indicate a positively perceived determinant of acceptability (promoting acceptability), while the revers holds true when both the mean and 95% confidence interval are below 3, indicating a negatively perceived determinant of acceptability (lowering acceptability).

The multidimensional “strength of acceptance” summary statistic indicates a high degree of acceptance (mean, 4.0; 95% CI, 3.7 to 4.3). The narrow confidence interval of the summary statistic results from the fact that a high degree of acceptance promoting user experience was documented for all determinants of acceptance: their mean scores range from 3.5 to 4.5, with largely overlapping confidence intervals. Differences between the determinants of acceptance were subtle, with somewhat lower scores for “perceived ease of use” (mean, 3.5; 95% CI, 3.0 to 4.0) and “social presence” (mean, 3.5; 95% CI, 2.8 to 4.1), while the highest scores were documented for “intention to use” (mean, 4.6; 95% CI, 4.1 to 5.0).

Part II presents data for the three more generally phrased acceptance items. Here, again, both the individual item’s statistics and the summary statistic (mean, 4.1; 95% CI, 3.3 to 4.9) indicated a high degree of acceptance (compare Table 4).

### 3.3. Multivariate Analysis of Modifiers of Acceptance

Multivariate general linear models, as described above, did not corroborate modifiers of either the multidimensional “strength of acceptance” statistic (F7,10 = 0.55; *p* = 0.7686) or the “general acceptance” statistic (F7,10 = 0.31; *p* = 0.9066). The following potential modifiers were tested: (1) age; (2) sex; (3) time post-stroke; (4) the syndrome treated, i.e., arm paresis or visual neglect; severity of stroke as impairment, including both (5) NIHSS scores and (6) syndrome assessment scores, i.e., FM Arm or NET; and (7) the BI as a measure of disability. None of these factors was statistically corroborated as a modifier of either the multidimensional “strength of acceptance” statistic (individual factors: F1,10 < 0.95; *p* > 0.4) or the “general acceptance” statistic (individual factors: F1,10 < 0.60; *p* > 0.5)

### 3.4. Narrative Summary of Answers to Usability Questions

Table 5 presents a narrative summary of the information gained by the responses of participants to the open questions (Part II, section b). Content summary statements are supplemented by a typical example of a response given, i.e., an original response and its English translation, in Table 5.

The clear speech of the humanoid robot and clear explanations, good visual presentations and speed, adequate training task selection, and number of repetitions, as well as the game-like approach, were perceived as strengths of the system (E-BRAiN). That explanations were perceived as lengthy and transition between steps was somewhat slow were reported as its weaknesses.

The following modifications were suggested: shorter explanations, the implementation of speech recognition as opposed to only a touchscreen interface, more specific guidance by the system when behavioural errors (by patients) occur, and more artificial intelligence to react to spontaneous behaviour by patients. Overall, the user experience was mostly considered positive. As further positive feature that was noted was that the robot does not get tired. It was considered helpful, but there was also concern that it would replace therapists.

## 4. Discussion

Over the last few decades, robotic technology for rehabilitation has received much attention in both engineering and clinical research and has promoted the development of systems that can effectively support rehabilitation for stroke survivors by the provision of mechanically assisted training [25].

Here, we report on a new and different technological approach to support rehabilitation post-stroke. By making use of humanoid robot technology and detailed algorithms to mimic human therapeutic interaction for a variety of effective training therapy schemas [11,12], a digital therapy system (E-BRAiN) was established that acts as a social agent and leads stroke survivors through their daily training sessions of prescribed therapy in an autonomous way [26].

The aim of the here-reported research was to evaluate technology acceptance and usability as perceived by stroke survivors who had experienced the use of the system for their own treatment.

Stroke survivors who participated in this research had a need for arm rehabilitation or neglect therapy (compare Table 3). The sample represented moderate severely affected stroke survivors receiving inpatient neurorehabilitation services in the sub-acute phase after stroke. The therapeutic setting was like other standard rehabilitation treatments available at medical facilities. Considering the characteristics of the study population and the therapeutic context, the study environment closely mirrored typical stroke rehabilitation scenarios, thus enhancing the ecological validity of the results. This implies that the findings are likely relevant to everyday clinical practice.

All participants received therapy lead by a humanoid robot over the course of two weeks with daily sessions (i.e., nine sessions led by the robot). Prior to the commencement of the robot-led therapy, a human therapist individualised the otherwise standardised and evidence-based training approaches as either arm rehabilitation therapy for strokes survivors with moderate-to-severe arm paresis, or neurovisual therapy for strokes survivors who had moderate-to-severe visual neglect, respectively [6,23,24].

Within the two weeks of experience with the humanoid robot administering their daily therapeutic sessions, these stroke survivors participated in a session where they had been asked to report about their personal experience of the robot therapy received thus far.

Two ways to assess technology acceptance were used in this research.

On one side, a comprehensive questionnaire with 41 items addressing different constructs that are related to technology acceptance in various areas of personal experience was used (compare Table 1, Table 2 and Table 4, Part I). This approach was based on the Almere model, which distinguishes several factors of personal experience that together influence technology acceptance and consequently modify the intention to use technology [15].

Overall, this multidimensional acceptance profiling indicated a high degree of acceptance of the E-BRAiN system by stroke survivors receiving their personal therapy as administrated by a humanoid robot (compare multidimensional “strengths of acceptance summary” statistic in Table 4, Part I). In more detail, the 41 items of the questionnaire reflected 12 different constructs that address personal experience regarding the used technology’s functionality, the social interaction aspects of a robot system, and the emotional experiences of the users. For those, it could be shown that, similarly to the overall strengths of acceptance, the individual constructs indicated personal experience that promotes a high degree of technology acceptance. Specifically, in terms of functionality, the users, on average, perceived the ability of the system to adapt to their needs (“perceived adaptiveness”), and they believed that the system is assistive (“perceived usefulness”) and perceived factors in the environment that facilitated its use, probably its embeddedness in the overall healthcare and therapy administration. Somewhat lower was the perception that using the system would be easy to use and could be used without any help (“perceived ease of use”). This is quite understandable since the system was set up and started by a therapist before the actual training with the robot as social agent and therapeutic assistant commenced. Aside from these aspects related to the system’s functionality, technology aspects that were related to the robot’s social interaction are covered by the questionnaire. The participants, on average, perceived an ability of the system to perform social behaviour (“perceived sociability”). They believed that the system performs with personal integrity and reliability (“trust”); they had—even though somewhat lower—the experience of sensing a social entity when interacting with the robot (“social presence”); and they rather had the perception that people who are important to them think that they should use the system (“social influence”). Finally, the questionnaire addresses emotional responses experienced by the users. The stroke survivors reported rather positive feelings about the appliance of technology (“attitude toward technology”), feelings of joy and pleasure associated with the use of the system (“perceived enjoyment”), but not anxious reactions when it comes to using the system (“anxiety”).

All of these various aspects of experience related to the systems functionality, the robot’s social interaction, and the feelings experienced while using the system reflected the same direction, i.e., promoting the users’ acceptance of the system. This translated to rather high scores on the “intention to use” item: the intention to use the system over a longer period of time was positively reported, with the highest scores among the items of the questionnaire (Part I).

In addition to the questionnaire’s part that was based on the Almere model (Part I), the second part of the questionnaire included three items for acceptance assessment that are more general, while at the same time specifically phrased for the E-BRAiN system (Part II, section a). These items were meant as an acceptance assessment independent of the Almere model. Here, again, for all three items and their average, the “general acceptance” scores a high degree of acceptance was documented (compare Table 4, Part II). The participants felt that the type of technology could well be used for stroke therapy, that it made sense to use the robot to supplement their own therapy, and that it was indeed fun to use it. Taken together, these more generally phrased items addressing acceptance were in good agreement with the multidimensional “strengths of assessment” profiling and can be considered a validating aspect, i.e., supporting the notion that technology acceptance was high.

Overall, the data suggest that the perceived functionality of the system, the implemented social behaviour of the humanoid robot, and the emotional reactions induced during therapeutic sessions as perceived by its users, as well as the perception that it makes sense to use the robot technology for stroke therapy and as a supplement for their own therapy, all supported a high degree of technology acceptance by stroke survivors and promoted their intention to use it.

The qualitative approach with open questions provided additional information about specific technological strengths and weaknesses and areas where the system could be further improved. Positive aspects such as a clear voice, clear explanations, and good visual presentations, as well as adequate training tasks and repetitions and the game-like approach that was fun, were reassuring. Users, however, felt that explanations provided by the robot could be kept somewhat shorter and that further developments of the system could integrate even more advanced technology, e.g., in terms of natural speech recognition or artificial intelligence to promote the system’s ability to more spontaneously react and interact with users. While the experience made with the humanoid robot was considered positive and helpful at the personal level, there was a concern that robots should not replace therapists.

These personal statements are similar to those previously noted by stroke survivors using an arm rehabilitation system employing a humanoid robot. The main disadvantages reported were that the robot did not possess human abilities, such as the ability to hold a conversation, to physically guide the patient’s movements, and to express or understand emotions [27]. While artificial intelligence (AI) applications have previously been shown to support a multitude of aspects, such as diagnostics and prediction, that are relevant for rehabilitation [28] and have certainly a potential to enhance rehabilitation technology, AI applications for the implementation of more “fluid” human abilities will still have to await further technological developments. In addition, while natural language processing (NLP) has been used for stroke management, this is so for medical report-related purposes, but not for stroke rehabilitation administration [29]. Indeed, speech and language impairments that frequently affect stroke survivors would pose specific challenges to do so [30]. Nevertheless, NLP and other AI algorithms to support more spontaneous interaction would certainly promote an adaptive and supportive social interaction and, hence, would be highly warranted.

The multivariate analyses of personal characteristics that could potentially modify technology acceptance did not corroborate relevant factors within the dataset. Hence, the positive acceptance profiling applies to all stroke survivors included independent of their personal characteristics.

The acceptance profiling is reassuring, especially since both the multidimensional and the more general approach convergently supported a high degree of technology acceptance for the system.

The reported findings might not be unexpected, as—prior to this research—the system was systematically developed to address stroke survivors’ rehabilitation needs appropriately (including in an acceptable way), comprehensively, and effectively.

As a stand-alone emerging technology, its value- and capability-sensitive design was based on clinical expert knowledge and experience using an anticipatory approach with the goal to develop a medically and psychosocially adequate therapeutic assistance with an expert system using a humanoid robot as social agent, and thereby to implement desired values in the technology and support individual and societal needs [31,32]. Specifically, the technology was designed to support users to regain capabilities lost by their stroke and promote their autonomy in everyday life and social participation. To promote a high clinical benefit, i.e., to promote the individual user’s capabilities, it implemented rehabilitation therapy of known effectiveness as suggested for clinical practice in international guidelines [7]. Its therapeutic interaction was based on research that specifically characterised the typical human therapeutic interactions for the therapeutic approaches implemented [11,12]. Furthermore, stroke survivors do have specific handicaps (e.g., cognitive, motor, or perceptual impairments) that add to the complexity of human–technology interaction. Hence, technology design was chosen to explicitly address the related needs of vulnerable people as users, e.g., by appropriately selected interaction, content, and framing of information provision, motivational support, and consideration of other needs (such as breaks). Last, but not least, prior to its current use in the clinical trial [18], the system had already reiteratively and incrementally been adapted to user needs based on expert reviews.

With this prior research and development background, it was then important to evaluate whether this patient-centred eHealth system development did result in a high degree of technology acceptance for prospective users [33]. Indeed, the participating users evaluated the technology positively; it seemed to adequately address their needs and preferences regarding the therapeutic support (functional acceptance), the adequacy of the robot’s social interaction (social acceptance), and their emotional needs and well-being. The data therefore support the notion of the achievement of an appropriate user-centred design.

The research findings agree with results of the limited research conducted in the domain. Previous systems have also shown that socially interactive humanoid robots can be effectively utilised for post-stroke arm rehabilitation in a clinically significant and acceptable manner for stroke survivors [9,10]. Stroke survivors receiving a training administered by a humanoid robot more frequently reported patients’ satisfaction with the rehabilitation activity, trust in the rehabilitation system’s functional skills, and its contribution to patients’ hand functions compared to those who received the same type of training administered by a computer only [9]; in addition, they achieved clinically relevant gains of their arm and hand function, while the control group did not [10]. Similarly, an immersive virtual-reality mirrored hand system for upper limb stroke rehabilitation, an alternative approach to support rehabilitation via technology, was shown to receive only moderately acceptable clinical usability ratings from stroke survivors (n = 15; System Usability Scale, SUS, scores range from 0 to 100: mean SUS score of 56.67; SD, 13.88; 3 users (20%) had score > 70, indicating a good usability) [34]. The limitation of the research is the size of the study population questioning its representativeness, especially for female users, who were underrepresented in the sample. Nevertheless, the multivariate analysis did not imply relevant modifiers based on personal characteristics supporting the data’s broader applicability. Further, the number of participants and, hence, the dataset are not sufficient to validate the constructs of the Almere model for the population of stroke survivors, e.g., by factor analysis. While noteworthy as a fact, this was not the intention of this research.

The research findings are nonetheless of more general importance: They add important information regarding the acceptance profile of the therapeutic system E-BRAiN for clinical users based on their experience and evaluation.

With the growing global demand for solutions addressing neuro-disabilities [1], technologies such as the one investigated here could play a crucial role in rehabilitative healthcare once they are proven to be both acceptable to individuals with neuro-disabilities needing rehabilitation (as demonstrated in this research), promote the achievement of therapeutic goals effectively, and are both clinically safe and cost-effective.

The unique advantages of humanoid robots in rehabilitation therapy compared to traditional rehabilitation equipment [25] is that humanoid robots that are capable of human-like therapeutic interaction [12] can provide a more personalised treatment experience, and they might thereby promote adherence to training schedules and, hence, support the intended clinical outcome while at the same time requiring less of the limited human therapeutic resources.

## Figures and Tables

**Table 1:** Explanation of assessed determinants of the intention to use technology.

Code	Construct	Definition
ANX	Anxiety	Evoking anxious or emotional reactions when it comes to using the system
ATT	Attitude	Positive or negative feelings about the appliance of the technology
FC	Facilitating	Factors in the environment that facilitate use of conditions the system
ITU	Intention to Use	The intention to use the system over a longer period in time
PAD	Perceived adaptiveness	The perceived ability of the system to adapt to the needs of the user
PENJ	Perceived Enjoyment	Feelings of joy/pleasure associated with the use of the system
PEOU	Perceived Ease of Use	The degree to which one believes that using the system would be free of effort
PS	Perceived Sociability	The perceived ability of the system to perform sociable behaviour
PU	Perceived Usefulness	The degree to which a person believes that the system would be assistive
SI	Social Influence	The person’s perception that people who are important to him/her think he/she should or should not use the system
SP	Social Presence	The experience of sensing a social entity when interacting with the system
Trust	Trust	The belief that the system performs with personal integrity and reliability

From Heerink et al. [15].

**Table 2:** Items used to assess determinants of intention to use technology.

Code	Item
ANX	1. If I should use the robot, I would be afraid to make mistakes with it
	2. If I should use the robot, I would be afraid to break something
	3. I find the robot scary
	4. I find the robot intimidating
ATT	5. I think it’s a good idea to use the robot
	6. The robot would make life more interesting
	7. It’s good to make use of the robot
FC	8. I have everything I need to use the robot
	9. I know enough of the robot to make good use of it
ITU	10. I think I’ll use the robot during the next few days
	11. I’m certain to use the robot during the next few days
	12. I plan to use the robot during the next few days
PAD	13. I think the robot can be adaptive to what I need
	14. I think the robot will only do what I need at that particular moment
	15. I think the robot will help me when I consider it to be necessary
PENJ	16. I enjoy the robot talking to me
	17. I enjoy doing things with the robot
	18. I find the robot enjoyable
	19. I find the robot fascinating
	20. I find the robot boring
PEOU	21. I think I will know quickly how to use the robot
	22. I find the robot easy to use
	23. I think I can use the robot without any help
	24. I think I can use the robot when there is someone around to help me
	25. I think I can use the robot when I have a good manual
PS	26. I consider the robot a pleasant conversational partner
	27. I find the robot pleasant to interact with
	28. I feel the robot understands me
	29. I think the robot is nice
PU	30. I think the robot is useful to me
	31. It would be convenient for me to have the robot
	32. I think the robot can help me with many things
SI	33. I think the staff would like me using the robot
	34. I think it would give a good impression if I should use the robot
SP	35. When interacting with the robot I felt like I’m talking to a real person
	36. It sometimes felt as if the robot was really looking at me
	37. I can imagine the robot to be a living creature
	38. I often think the robot is not a real person
	39. Sometimes the robot seems to have real feelings
Trust	40. I would trust the robot if it gave me advice
	41. I would follow the advice the robot gives me

From Heerink et al. [15].

**Table 3:** Study population characteristics (n = 11).

	Mean/SD	Min–Max	
	n (%)	n (%)	n (%)
**Age** (mean/SD, min–max)	62.3/8.9	49–77	
**Sex** (male, female) (n (%))	9 (82%)	2 (18%)	
**Stroke type** (ischemic, ICH) (n (%))	11 (100%)	0 (0%)	
**Affected brain** (left, right) (n (%))	1 (9%)	10 (91%)	
**Time post-stroke (weeks)** (mean/SD, min–max)	4.9/1.6	3–8	
**NIHSS** (0–42) (mean/SD, min–max)	8.7/3.5	4–15	
**Barthel Index** (0–100) (mean/SD, min–max)	61/20	35–95	
**FM Arm **^a^ (0–66) (mean/SD, min–max; n)	22.8/11.7	11–40	5
**NET **^b^ (0–160) (mean/SD, min–max; n)	110.4/28.6	60.5–146.5	6
**Type of training therapy** (ABT ^a^,^,^ MT ^a^, NT ^b^)	4 (36%)	1 (9%)	6 (55%)

Abbreviations: ABT—arm basis training; FM Arm—Fugl–Meyer Arm Motor score; ICH—intracerebral haemorrhage; NIHSS—National Institute of Health Stroke Scale; NET—Neglect Test; NT—neglect therapy; MT—mirror therapy; min—minimum; max—maximum; SD—standard deviation. Superscript letters (ABT ^a^/MT ^a^, and NT ^b^) indicate the different types of therapy and how they relate to both the treated syndromes and the tests used for baseline assessment, respectively (i.e., arm paresis/FM Arm a and visual neglect/NET b).

**Table 4:** Acceptability profile (n = 11).

Code	Description	Mean [95% CI]	
*Part I*			
ANX	Anxiety	4.5	[4.1–5.0]
ATT	Attitude toward technology	4.5	[4.1–4.8]
FC	Facilitating conditions	3.9	[3.1–4.7]
ITU	Intention to use	4.6	[4.1–5.0]
PAD	Perceived adaptiveness	3.9	[3.3–4.5]
PENJ	Perceived enjoyment	4.1	[3.7–4.6]
PEOU	Perceived ease of use	3.5	[3.0–4.0]
PS	Perceived sociability	3.9	[3.2–4.6]
PU	Perceived usefulness	3.9	[3.3–4.5]
SI	Social influence	4.0	[3.4–4.6]
SP	Social presence	3.5	[2.8–4.1]
Trust	Trust	4.1	[3.5–4.7]
SACC	Strength of acceptance	4.0	[3.7–4.3]
*Part II*			
Gen1	Robot for stroke	4.5	[3.9–5.0]
Gen2	Robot therapy for me	4.5	[4.1–5.0]
Gen3	Fun	4.1	[3.5–4.7]
GenAVG	General acceptance	4.1	[3.3–4.9]

Abbreviations/explanations: 95% CI–95% confidence interval. Part I: Given are average statistics across items that individually range from 1 to 5 (low-to-high acceptability; with a score of 3 being neutral) and belong to a certain construct as listed (and further explained in Table 1 and Table 2); SACC—average score for all other construct statistics of Part I. Part II: Gen1, Gen2, and Gen3—three more generally phrased Likert-scale items, i.e., (1) “I think it’s a good idea to use a robot in stroke therapy” (“robot for stroke”); (2) “I think it makes sense to use this therapy with the robot as a supplement to my therapy” (“robot therapy for me”); and (3) “A therapy session with the robot is fun” (“fun”). GenAVG—average score for Gen1, Gen2, and Gen3.

**Table 5:** Narrative summary of answers to usability questions.

*Strengths of the system*		
Clear speech and explanations	“Er spricht deutlich, laut. Aufgabeerklärung deutlich”.	“He speaks clearly, loudly. Task explanation clearly”.
Good visual presentations and speed.	“alles gut sehbar, Tempo und Farbe gut”.	“Everything easy to see, good speed and colour”.
Adequate training tasks and repetitions.	“Die Übungen waren gut”. “Die Wiederholung ist wichtig”.	“The exercises were good”. “Repetition is important”.
Game-like approach (fun)	“Das spielerische Herangehen war gut”.	“The playful approach was good”.
*Weaknesses of the system*		
Explanations lengthy	“Der Roboter redet lange”.	“The robot talked for a long time”.
Transition between steps somewhat slowly	“Etwas langsam, schneller schalten”.	“Somewhat slowly, quicker changes”.
*Areas where modifications were suggested by users*		
Shorter explanations	“Er könnte sich etwas kürzer fassen”.	“He could be a bit shorter”.
Speech recognition as opposed to touch screen interface	“Spracherkennung, statt der Tasten-Funktion”.	“Voice recognition instead of the button function”.
More specific guidance when behavioural errors (patient) occur	“Den Fehler alleine aufzeigen reicht nicht, sondern müssen auch zeigen, wo der Fehler ist und wie man besser wird”.	“Simply pointing out the error is not enough, you also have to show where the error is and how you can improve”.
More artificial intelligence to react to spontaneous utterances by patients	“Mehr Intelligenz, was gesagt wird als Patient, auf Bedürfnisse automatisch reagieren können”.	“More intelligence, what is said as a patient, being able to react automatically to needs”.
*Overall users’ subjective experience*		
Mostly positive	“Alles super, ich bin zufrieden”.	“Everything is great, I’m satisfied”.
Robot does not get tired	“Er ermüdet nicht”.	“He doesn’t get tired”.
Helpful, but should not replace therapists	“In meinem Fall hilfreich, aber ich will nicht, dass er es ersetzt”.	“Helpful in my case, but I don’t want it to replace it.”

Explanations: Presented is a narrative summary of the information gained by the responses of participants (n = 11) to the open question (i = 5) (Part II, section b). Content summary statements (left column) are supplemented by a typical example of response given, i.e., an original response (middle column) and its English translation (right column).

## Data Availability

The datasets presented in this article are not readily available because any use of the data is restricted to the purposes and for the persons explicitly declared in the written informed consent. Any request regarding data availability should be addressed to the first author (T.P.).

## Supplementary Materials

The following supporting information can be downloaded at https://www.mdpi.com/article/10.3390/biomimetics10050289/s1, Original 5-page questionnaire (in German).
