# Supplementary material for: Technology Acceptance and Usability of a Therapy System with a Humanoid Robot Serving as Therapeutic Assistant for Post-Stroke Arm and Neurovisual Rehabilitation—An Evaluation Based on Stroke Survivors’ Experience
Source: Biomimetics (Basel). 2025 May 4;10(5):289. doi: 10.3390/biomimetics10050289 (PMC12108900; doi:10.3390/biomimetics10050289)
Supplement: Supplementary file 1 [file biomimetics-10-00289-s001.zip › biomimetics-3546293-supplementary.pdf]

E·BRAiN

Fragebogen zur Einschätzung des Therapiesystems durch Nutzer

Sehr geehrte E-BRAiN Studienteilnehmerin, sehr geehrter Studienteilnehmer,

vielen Dank, dass Sie sich die Zeit nehmen, diesen Fragebogen auszufüllen. Die Ergebnisse dieses Fragebogens werden dabei helfen, den Roboter und die E-BRAiN-Therapie weiter zu verbessern.

Das E-BRAiN System wurde entwickelt, um Therapien für Schlaganfall-Betroffene mit einem humanoiden Roboter als Therapieassistenten zu unterstützen. Wir sind weltweit die erste Langzeitstudie mit Robotern für diese Art von Therapien. Auch wenn wir uns die größtmögliche Mühe gegeben haben, damit Sie eine erfolgreiche Therapie haben, gibt es immer Punkte, die wir besser machen können.

Ihr Feedback werden wir nutzen, um die nächste Version des Systems zu verbessern und die Therapiesitzung für Patienten angenehmer zu gestalten. Diese Verbesserungen werden auch dabei helfen, die Patienten dazu zu motivieren, die Therapie weiterzuverfolgen - jede absolvierte Therapiestunde hilft auf dem Weg der Rehabilitation.

Ihr Feedback ist uns also sehr wichtig!

1. Datum, Personenidentifikation und durchgeführte Therapie

Datum:

Personenidentifikation (Studiennummer):

Durchgeführte Therapie:

Hinweis: In den Fragen werden Aussagen über „**den Roboter**“ und „**das System**“ gemacht. Hiermit meinen wir das **gesamte E-BRAiN Therapiesystem**, den **Roboter,** das **Tablet** und die **anderen Komponenten**, mit denen Sie die Therapiesitzungen absolviert haben.

2. Fragen zum Roboter (nach Heerink et al., 2010; deutsche Übersetzung A. Bundea & T. Platz)

	Stimme überhaupt nicht zu	Stimme nicht zu	Ich weiß nicht	Stimme zu	Stimme komplett zu
Wenn ich den Roboter benutzen sollte, hätte ich Angst, mit diesem Fehler zu machen.	☐	☐	☐	☐	☐
Wenn ich den Roboter benutze sollte, hätte ich Angst, etwas zu beschädigen.	☐	☐	☐	☐	☐
Ich finde den Roboter gruselig.	☐	☐	☐	☐	☐
Ich finde den Roboter einschüchternd.	☐	☐	☐	☐	☐
Ich denke, es ist eine gute Idee, den Roboter zu nutzen.	☐	☐	☐	☐	☐
Der Roboter würde das Leben interessanter machen.	☐	☐	☐	☐	☐
Es ist gut, den Roboter zu nutzen.	☐	☐	☐	☐	☐
Ich habe alles, um den Roboter zu nutzen.	☐	☐	☐	☐	☐
Ich weiß genug über den Roboter, um ihn benutzen zu können.	☐	☐	☐	☐	☐
Ich denke, ich werde den Roboter in den nächsten Tagen verwenden.	☐	☐	☐	☐	☐
Ich werde den Roboter sicher in den nächsten Tagen verwenden.	☐	☐	☐	☐	☐
Ich plane, den Roboter in den nächsten Tagen zu verwenden.	☐	☐	☐	☐	☐
Ich denke, der Roboter kann sich an das anpassen, was ich brauche.	☐	☐	☐	☐	☐
Ich denke, der Roboter wird nur das tun, was ich für diesen bestimmten Moment brauche.	☐	☐	☐	☐	☐
Ich denke, der Roboter wird mir helfen, wenn ich es für notwendig halte.	☐	☐	☐	☐	☐
Ich genieße es, wenn der Roboter zu mir spricht.	☐	☐	☐	☐	☐
Ich mache gerne Dinge mit dem Roboter.	☐	☐	☐	☐	☐
Ich finde den Roboter angenehm.	☐	☐	☐	☐	☐
Ich finde den Roboter faszinierend.	☐	☐	☐	☐	☐
Ich finde den Roboter langweilig.	☐	☐	☐	☐	☐
Ich denke, ich werde schnell wissen, wie man den Roboter benutzt.	☐	☐	☐	☐	☐
Ich finde der Roboter ist einfach zu benutzen.	☐	☐	☐	☐	☐
Ich denke, ich kann den Roboter ohne Hilfe benutzen.	☐	☐	☐	☐	☐

3. Fragen zum Roboter (Fortsetzung) (nach Heerink et al., 2010; deutsche Übersetzung A. Bundea & T. Platz)

	Stimme überhaupt nicht zu	Stimme nicht zu	Ich weiß nicht	Stimme zu	Stimme komplett zu
Ich denke, ich kann den Roboter benutzen, wenn jemand da ist, um mir zu helfen.	☐	☐	☐	☐	☐
Ich denke, ich kann den Roboter benutzen, wenn ich eine gute Gebrauchsanweisung habe.	☐	☐	☐	☐	☐
Ich halte den Roboter für einen angenehmen Gesprächspartner.	☐	☐	☐	☐	☐
Ich finde es angenehm, mit dem Roboter zu interagieren.	☐	☐	☐	☐	☐
Ich habe das Gefühl, der Roboter versteht mich.	☐	☐	☐	☐	☐
Ich denke, der Roboter ist nett.	☐	☐	☐	☐	☐
Ich denke, der Roboter ist nützlich für mich.	☐	☐	☐	☐	☐
Es wäre praktisch für mich, den Roboter zu haben.	☐	☐	☐	☐	☐
Ich denke, der Roboter kann mir bei vielen Dingen helfen.	☐	☐	☐	☐	☐
Ich glaube, das Personal möchte, dass ich den Roboter benutze.	☐	☐	☐	☐	☐
Ich denke, es würde einen guten Eindruck machen, wenn ich den Roboter verwende.	☐	☐	☐	☐	☐
Als ich mit dem Roboter interagierte, fühlte ich mich so, als ob ich mit einer realen Person sprechen würde.	☐	☐	☐	☐	☐
Manchmal fühlte es sich an, als ob der Roboter mich wirklich ansieht.	☐	☐	☐	☐	☐
Ich kann mir vorstellen, dass der Roboter ein Lebewesen ist.	☐	☐	☐	☐	☐
Ich denke oft, dass der Roboter keine echte Person ist.	☐	☐	☐	☐	☐
Manchmal scheint der Roboter echte Gefühle zu haben.	☐	☐	☐	☐	☐
Ich würde dem Roboter vertrauen, wenn er mir Ratschläge geben würde.	☐	☐	☐	☐	☐
Ich würde dem Rat folgen, den der Roboter mir gibt.	☐	☐	☐	☐	☐

4. Allgemeines zum System

	Stimme überhaupt nicht zu	Stimme nicht zu	Ich weiß nicht	Stimme zu	Stimme komplett zu
Ich denke, es ist eine gute Idee, einen Roboter in der Schlaganfalltherapie einzusetzen.	☐	☐	☐	☐	☐
Ich denke, es macht Sinn, diese Therapie mit dem Roboter als Ergänzung zu meiner Therapie einzusetzen.	☐	☐	☐	☐	☐
Eine Therapiesitzung mit dem Roboter macht Spaß.	☐	☐	☐	☐	☐

5. Offene Fragen

Was hat Ihnen bei diesem System (und Ihrer Therapie) am besten gefallen?

Was hat Ihnen an diesem System (und Ihrer Therapie) am wenigsten gefallen?

Wenn Sie eine Sache an diesem System ändern oder hinzufügen könnten, was wäre das?

Bitte denken Sie nun über Ihre Erfahrung mit dem E-BRAiN System insgesamt nach:<br>Wie fanden Sie die Therapie mit dem Roboter?<br>Was denken Sie über den Roboter als Therapeuten?

6. Persönliches Feedback (Freitext)

Gibt es etwas, was Sie uns noch über das System sagen wollten?

Vielen Dank für Ihre Beantwortung unserer Fragen**!**
